# Coordinated Approach Fusing RCMDE and Sparrow Search Algorithm-Based SVM for Fault Diagnosis of Rolling Bearings

**DOI:** 10.3390/s21165297

**Published:** 2021-08-05

**Authors:** Jie Lv, Wenlei Sun, Hongwei Wang, Fan Zhang

**Affiliations:** School of Mechanical Engineering, Xinjiang University, Urumqi 830047, China; lvjie@stu.xju.edu.cn (J.L.); wanghongwei@stu.xju.edu.cn (H.W.); zhangfan@stu.xju.edu.cn (F.Z.)

**Keywords:** fault diagnosis, variational mode decomposition (VMD), refined composite multiscale dispersion entropy (RCMDE), sparrow search algorithm (SSA), support vector machine (SVM)

## Abstract

We propose a novel fault-diagnosis approach for rolling bearings by integrating variational mode decomposition (VMD), refined composite multiscale dispersion entropy (RCMDE), and support vector machine (SVM) optimized by a sparrow search algorithm (SSA). Firstly, VMD was selected from various signal decomposition methods to decompose the original signal. Then, the signal features were extracted by RCMDE as the input of the diagnosis model. Compared with multiscale sample entropy (MSE) and multiscale dispersion entropy (MDE), RCMDE proved to be superior. Afterwards, SSA was used to search the optimal parameters of SVM to identify different faults. Finally, the proposed coordinated VMD–RCMDE–SSA–SVM approach was verified and evaluated by the experimental data collected by the wind turbine drivetrain diagnostics simulator (WTDS). The results of the experiments demonstrate that the proposed approach not only identifies bearing fault types quickly and effectively but also achieves better performance than other comparative methods.

## 1. Introduction

Rolling bearings are widely used in transmissions, generators, machine tools, and other high-speed rotating machinery. Once the bearing fails, the performance of the rotating mechanism will be affected [1]. The operational environment of the bearing is complex, and various types of faults can easily appear in the long-term work, causing potential danger to the mechanical operations [2]. However, in the field of bearing fault diagnosis, many similar algorithms have the disadvantages of low classification accuracy and long calculation time. These shortcomings lead to the poor effect of fault diagnosis in practical application. How to identify fault types quickly and effectively has become an important topic. Therefore, this paper proposes a new bearing-fault diagnosis method, which integrates several algorithms to help quickly detect bearing faults and reduce losses. This paper obtains fault information from the vibrational signals of rolling bearings [3,4]: feature extraction and fault classification.

Feature extraction is a way to show the representative fault information in the form of a feature vector. Only by selecting effective feature extraction methods and improving the quality of feature set can a good fault-classification effect be achieved [5]. Selecting an effective feature-extraction method can improve the quality of feature set and achieve a good fault classification effect. Bearings have operated in complex working conditions for a long time, and the fault features in the original vibration signal collected are often submerged by noise and redundant signals, which cause great interference relative to fault diagnosis. Meanwhile, the fault-vibration signals of rolling bearings have nonstationary and nonlinear characteristics [6]. To solve these problems, many scholars have developed many solutions. Li [7] applies modified ensemble empirical mode decomposition (MEEMD) to feature extraction. Although MEEMD reduces the influence of noise in ensemble empirical mode decomposition (EEMD), it needs further processing to reduce the number of pseudo components. Smith [8] proposed another adaptive signal decomposition method called local mean decomposition (LMD). However, it has the problems of large amounts of iterative calculations and endpoint effects [9]. The variational mode decomposition (VMD) method proposed by Dragomiretskiy [10] not only deals with nonlinear and nonstationary signals effectively, but also suppresses modal aliasing and endpoint effects effectively, and the performance of signal decomposition is better. Therefore, this work selected VMD to process the original vibration signal.

The vibration-signal data obtained by VMD decomposition needs further processing to extract effective fault-feature information. A series of feature-extraction methods based on entropy are widely used in the field of fault diagnosis, such as sample entropy (SE) [11], permutation entropy (PE) [12], and fuzzy entropy (FE) [13]. However, the above methods are based on single-scale analysis of time series and cannot reflect complex features. Therefore, scholars have proposed the corresponding multiscale analysis method. Azami et al. [14] proposed enhanced multiscale permutation entropy (EMPE) to compensate for the deficiencies of MPE in the coarsening process. Zheng et al. [15] successfully applied multiscale sample entropy (MSE) to rolling-bearing-fault diagnosis, but MSE has high time cost and is easily affected by mutation signals. To overcome the defects of the above methods, M. Rostaghi et al. [16] proposed the dispersion entropy (DE) method. Through experimental comparison, DE has faster operational speed and higher stability. Based on this, Azami et al. [17] proposed the refined composite multiscale dispersion entropy (RCMDE) method. Compared with MSE and other methods, it was found that MDE and RCMDE had faster calculation speeds and better stability than MDE for noisy signals. Therefore, RCMDE was selected as the bearing-fault feature-extraction scheme.

After obtaining the fault feature vectors, to improve the classification accuracy and calculation speed, in this work we selected a support vector machine (SVM) as the classifier. The key step of the SVM algorithm is to determine the optimal values of penalty factor c and kernel parameter σ. The swarm-intelligence optimization algorithm achieves a global optimization effect by simulating the behavior of social animals. Zhang et al. [18] used particle swarm optimization (PSO) to optimize the parameters of SVM, which improved the fault-recognition rate. Compared with PSO, the Beetle Antennae search algorithm (BAS) used in reference [19] shows faster calculation speed and does not fall easily into the local optimal solution. The results show that the fault-recognition accuracy of the salp swarm optimization support vector machine (SSO-SVM) in reference [20] reaches 100%, which is superior to PSO and the grey wolf optimizer (GWO). Inspired by the group behavior of sparrows, Xue et al. [21] proposed the SSA, which has strong optimization ability and fast convergence speed. In this paper, SSA was selected for parameter optimization of SVM.

In summary, the main contributions of this paper are as follows. First, a fault feature-extraction method based on VMD and RCMDE is proposed to construct feature vectors for training and testing. Second, the SSA is applied to optimize the SVM, and the rolling-bearing-fault classifier is constructed. Last, this paper proposes a rolling-bearing-fault diagnosis model based on VMD, RCMDE, and SSA-optimized SVM. Experimental results show that the fault-diagnosis accuracy of the proposed method is 100%, and the classification efficiency is better than that of other similar methods. At the same time, the introduction of SSA in this paper will help other scholars better to understand the swarm-intelligence algorithm.

## 2. Methodology

### 2.1. Variational Mode Decomposition

This work selects VMD to process the original vibration signal. The center frequency and bandwidth of each component are determined by iteratively searching the optimal solution of the variational model, so as to realize the frequency domain division of the signal and the effective separation of each component. The calculation processes of VMD are as follows.

By introducing the quadratic penalty factor c and Lagrange multiplication operator, the constrained variational problem is transformed into an unconstrained variational problem. The extended Lagrangian expression is as follows:

(1) $$\begin{array}{l} L(\{u_{k}\},\{\omega_{k}\},\lambda )=\\ \alpha {\sum_{k}\left\|\partial_{t}\left(\delta (t)+\frac{j}{\pi t}\right)u_{k}(t)e^{-jw_{k}t}\right\|}_{2}^{2}+{\left\|f(t)-\sum_{k}u_{k}(t)\right\|}_{2}^{2}+\langle \lambda (t),f(t)-\sum_{k}u_{k}(t)\rangle \end{array}$$

It uses ADMM to calculate the best solution to the augmented Lagrangian function and uses VMD to calculate the original signal into k narrow-band intrinsic mode function components [5]. The VMD algorithm steps:

(1) Initialization parameters $\left\{u_{k}^{1}\right\}$, $\left\{\omega_{k}^{1}\right\}$, ${\hat{\theta }}^{1}$, and *n*, *n =* 0;
(2) *n* = *n* + 1, start the cycle;
(3) Update the spectrum of each mode according to the following formula:
  (2) $${\hat{u}}_{k}^{n+1}(\omega )=\frac{\hat{f}(\omega )-\sum_{k}{\hat{u}}_{k}(\omega )+\frac{\hat{\theta }(\omega )}{2}}{1+2C{\left(\omega -\omega_{k}\right)}^{2}},$$
(4) Update the center frequency:
  (3) $$\omega_{k}^{n+1}=\frac{\int_{0}^{\infty }\omega {\left|{\hat{u}}_{k}(\omega )\right|}^{2}d\omega }{\int_{0}^{\infty }{\left|{\hat{u}}_{k}(\omega )\right|}^{2}d\omega },$$
(5) Update the Lagrange multiplier as follows:
  (4) $${\hat{\theta }}^{n+1}(\omega )\leftarrow {\hat{\theta }}^{n}(\omega )\leftarrow \tau \left[\hat{f}(\omega )-\sum_{k}{\hat{u}}_{k}^{n+1}(\omega )\right],$$
  where the parameter *τ* is used to update the Lagrange multiplier.
(6) For the given discrimination accuracy, repeat steps (2)–(5) and *e* > 0 until the iterative condition is satisfied:
  (5) $$\frac{{\sum_{k}\left\|{\hat{u}}_{k}^{n+1}-{\hat{u}}_{k}^{n}\right\|}_{2}^{2}}{{\left\|{\hat{u}}_{k}^{n}\right\|}_{2}^{2}}<e,$$

### 2.2. Refined Composite Multiscale Dispersion Entropy

The vibration-signal data obtained by VMD decomposition needs further processing to extract effective fault-feature information. In this work, RCMDE was selected to extract feature vectors. Then, the obtained feature vectors were used as samples for training and testing. The solution processes of RCMDE are as follows.

Dispersion entropy (DE) is a nonlinear dynamic method used to characterize the complexity and degree of irregularity of time series. Its calculation method is detailed in reference [22]. Similar to the MDE, RCMDE also scales the raw data, but the difference is that MDE divides data isometrically and then averages them, while RCMDE refines them on the basis of MDE. First, the raw data is divided into segments with *τ* length from (1, *τ*)—several different initial points. The average value of each segment is calculated, and then these average values are arranged in order as a coarse-grained sequence. A total of *τ* coarse-grained sequences were obtained. Then the probability of each coarse-grained sequence’s distribution pattern is calculated, and the average of the probability is calculated. Finally, RCMDE is calculated according to Equation (7) [23]. RCMDE not only reduces the loss of information in the coarse-grained process of the MDE algorithm but also effectively solves the influence of the initial point position on the signal-processing results.

According to the analysis of reference [16], this paper sets the RCMDE parameters as: embedding dimension *m* = 3, the number of classes *c* = 6, and time delay *d* = 1. The appropriate scale was selected according to the needs of the experimental analysis.

The RCMDE algorithm steps:

For the raw data, the *k*th coarse-grained sequence $x_{k}^{\left(\tau \right)}=\{x_{k,1}^{\left(\tau \right)},x_{k,2}^{\left(\tau \right)},\ldots \}$ is given by the following formula:

(6) $$\{\begin{array}{c} x_{k,j}^{\left(\tau \right)}=\frac{1}{\tau }\sum_{b=k+\tau (j-1)}^{k+j\tau -1}u_{b}\\ j=1,2,\ldots ,L/\tau \\ k=1,2,\ldots ,\tau , \end{array}$$

For each scale *τ*, the RCMDE entropy is defined as follows:

(7) $$\{\begin{array}{c} \begin{array}{l} E_{\left(RCMDE\right)}(X,m,c,d,\tau )=\\ -\sum_{\pi =1}^{c^{m}}\bar{P}(\pi_{v_{0}v_{1}\ldots v_{m-1}})\ln(\bar{P}(\pi_{v_{0}v_{1}\ldots v_{m-1}})) \end{array}\\ \bar{P}(\pi_{v_{0}v_{1}\ldots v_{m-1}})=\frac{1}{\tau }\sum_{k=1}^{\tau }P_{k}{}^{\left(t\right)} \end{array},$$

where $\bar{P}(\pi_{v_{0}v_{1}\ldots v_{m-1}})$ is the average probability of the distribution pattern of the coarse-grained sequence.

### 2.3. Sparrow Search Algorithm

As a kind of swarm-intelligence optimization algorithm, SSA has strong optimization ability and fast convergence speed. SSA simulates the sparrow’s foraging process. As a social animal, sparrows have an efficient division of labor within the population. Some sparrows, as discoverers, are responsible for discovering food-rich areas and providing guidance for other sparrows. Another grouping is the participants, who are sent a signal by the discoverer to find food and bring it home. The poorer the fitness of each sparrow, the hungrier the sparrows are, and the more they need to go to other places for food. The initial position of the watchman is randomly generated in the population. They can warn of the dangers and decide whether the population wants to give up food. Among them, discoverers and participants can exchange identities. When a participant finds a better source of food, it can change from a participant to a discoverer. However, the proportion of sparrows of each status in the population remains unchanged. At the same time, to respond to the danger in time, the population will randomly select an appropriate proportion of sparrows as watchmen to monitor and remind the population to adjust the search strategy and quickly move closer to the safe area [24]. The optimal parameters of SSA are penalty parameter c and kernel function parameter σ in SVM. The fitness function is the prediction accuracy of SVM.

The sparrows’ positions are represented by the following matrix:

(8) $$X=\left[\begin{array}{cccc} x_{1,1} & x_{1,2} & \ldots & x_{1,d}\\ x_{2,1} & x_{2,2} & \ldots & x_{2,d}\\ \ldots & \ldots & \ldots & \ldots \\ x_{n,1} & x_{n,2} & \ldots & x_{n,d} \end{array}\right],\;$$

where *n* is the sparrow number and *d* is the dimension of the variable to be optimized. The fitness values of sparrows are represented by the following vectors:

(9) $$F_{X}=\left[\begin{array}{c} f(\left[\begin{array}{cccc} x_{1,1} & x_{1,2} & \ldots & x_{1,d} \end{array}\right])\\ f(\left[\begin{array}{cccc} x_{2,1} & x_{2,2} & \ldots & x_{2,d} \end{array}\right])\\ \ldots \\ f(\left[\begin{array}{cccc} x_{n,1} & x_{n,2} & \ldots & x_{n,d} \end{array}\right]) \end{array}\right],\;$$

The discoverer updates the location as follows:

(10) $$x_{ij}^{t+1}=\{\begin{array}{l} x_{ij}^{t}\cdot \exp(\frac{-i}{\alpha \cdot iter_{\max}}),R_{2}<ST\\ x_{ij}^{t}+QL,R_{2}\geq ST\; \end{array},\;$$

where *iter_max_* is the maximum number of iterations; *α* is a uniform random number between (0,1]; *Q* is a random number that obeys the standard normal distribution; *L* is a matrix of 1 × *d* whose elements are all 1; *R*_2_ and *ST* represent the alarm value and the safety threshold, respectively.

The position update formula for the participant is described as follows:

(11) $$x_{ij}^{t+1}=\{\begin{array}{c} Q\cdot \exp(\frac{x_{wj}^{t}-x_{ij}^{t}}{i^{2}}),i>NP/2\\ x_{pj}^{t+1}+|x_{ij}^{t}-x_{pj}^{t+1}|,A^{+}L,otherwise \end{array},\;$$

where $x_{pj}^{t}$ is the best location of the discoverer at iteration *t* + 1; $x_{wj}^{t}$ denotes the current global worst location at iteration *t*; *NP* is the population size; and *A* represents a matrix of 1×*d* for which each element inside is randomly assigned 1 or −1, and $A^{+}=A^{T}{\left(AA^{T}\right)}^{-1}$.

The watchman updates the position as follows:

(12) $$x_{ij}^{t+1}=\{\begin{array}{c} x_{ij}^{t}+\beta |x_{ij}^{t}-x_{bj}^{t}|,f_{i}\neq f_{g}\\ x_{pj}^{t}+K\frac{\left|x_{ij}^{t}-x_{wj}^{t}\right|}{(f_{i}-f_{w})+\varepsilon },f_{i}=f_{g} \end{array},\;$$

where $x_{bj}^{t}$ is the global optimal position in the *t*th iteration; *β*, as the step-size control parameter, is a normal distribution of random numbers with a mean value of 0 and a variance of 1; *K*∈[−1, 1] is a random number; $f_{i}$ is the fitness value of the present sparrow; $f_{g}$ and $f_{w}$ are the current global best and worst fitness values, respectively.

The specific steps of the SSA are as follows:

(1) Initialize the sparrow population. Define the algorithm parameters and maximum number of iterations.
(2) The fitness values of the initial population are calculated and sorted.
(3) Use formulas (5)–(7) to update the locations of discoverers, participants, and watchmen.
(4) Obtain the current optimal value, if the iteration effect is better, then update. Then repeat steps 2 to 6 until the maximum number of iterations is reached.
(5) The global optimal value and optimal fitness value are outputted.

## 3. Proposed Approach

**Figure 1:**
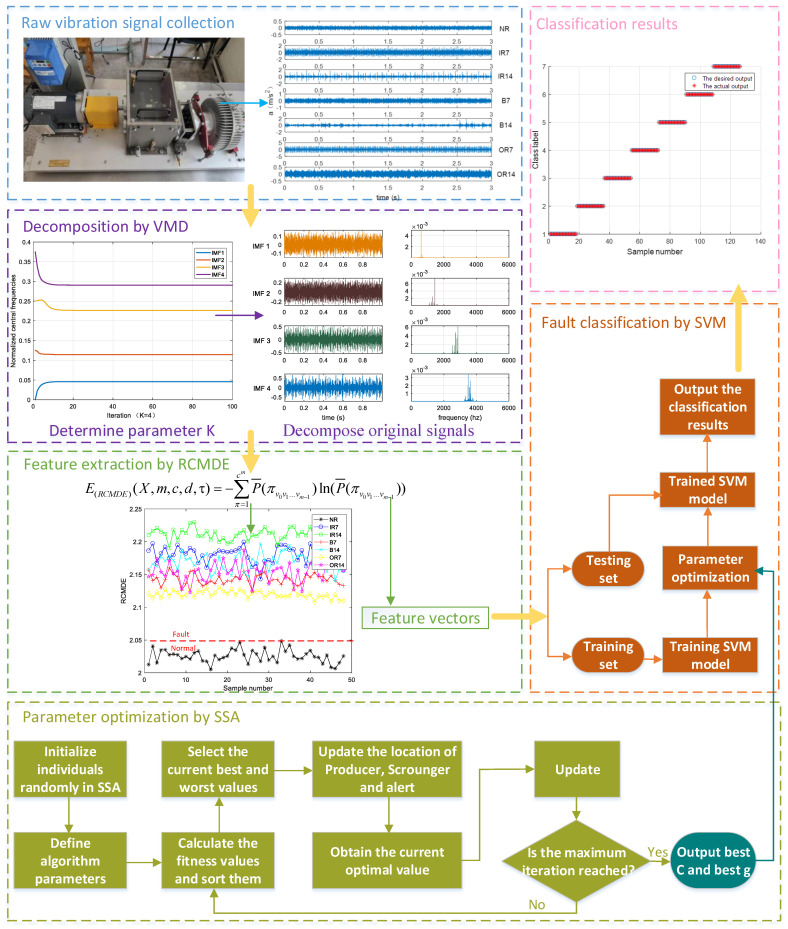
Technical roadmap of bearing-fault diagnosis.

Based on the above research, we propose a novel fault-diagnosis approach for rolling bearings by integrating VMD, RCMDE, and SVM optimized by SSA. The technical roadmap of fault classification is shown in Figure 1. The fault-classification sequence is as follows.

(1) The *k* parameter of VMD is selected, and then the original vibration signal is decomposed into *k* intrinsic mode functions (IMFs) by VMD.
(2) The best IMF component is selected.
(3) The best IMF components are grouped and the RCMDE values of each group are calculated. The appropriate scale feature vectors are selected to represent the fault features.
(4) The sparrow search algorithm is used to optimize the penalty factor *c* and kernel parameters *σ* of SVM.
(5) The extracted fault feature vectors are inputted into the classifier for training.
(6) The test set is inputted into the trained classifier for fault classification to verify the effectiveness of the proposed method.

## 4. Experiment and Discussion

### 4.1. Experimental Data

The experimental data of this study consists of two parts. One part is from the bearing database of Case Western Reserve University [25], which was used to select the parameters of the algorithm and establish the diagnosis model. The other part of the experimental data was collected from the WTDS in the laboratory to verify the effectiveness of the algorithm.

**Figure 2:**
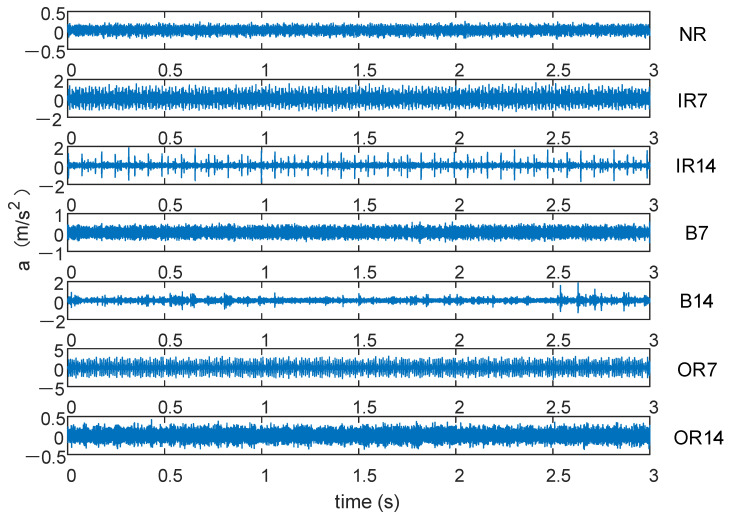
Original vibration signal.

For the bearing database of Case Western Reserve University, the driving-end fault data of the 6205-2RSJEMSKF deep-groove ball bearing is selected, where the fault diameters are 0.007 inch and 0.014 inch, respectively; the motor speed is 1750 rpm; the sampling frequency is 12 kHz; and the sampling time is 8 s. The vibration data of bearings under seven working conditions were collected, including one healthy bearing and six fault bearings. The fault types include inner race fault, outer race fault, and ball fault. Forty-eight groups of data were collected in each state, and each group contained 2000 sampling points. The detailed parameters of the bearing are shown in Table 1. The original vibration signal of the first 3 s is shown in Figure 2. For these seven kinds of fault data, we give them different classification labels according to different properties of bearings: NR, IR7, IR14, B7, B14, OR7, OR14.

### 4.2. Signal Decomposition by VMD

**Figure 3:**
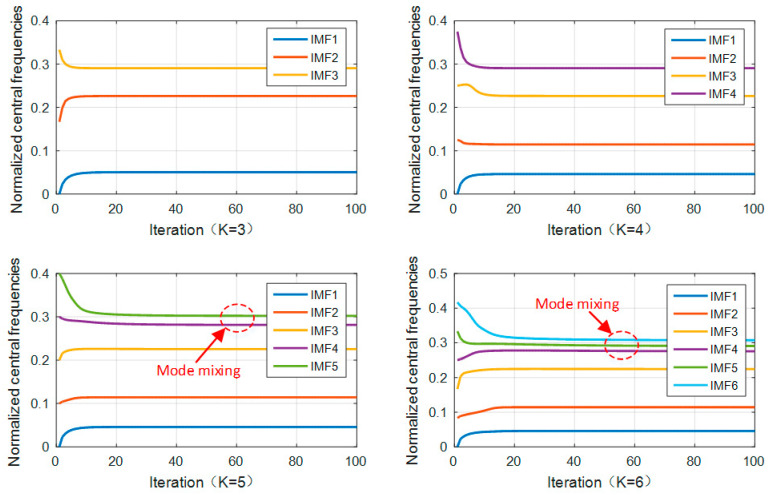
Central frequency curves.

**Figure 4:**
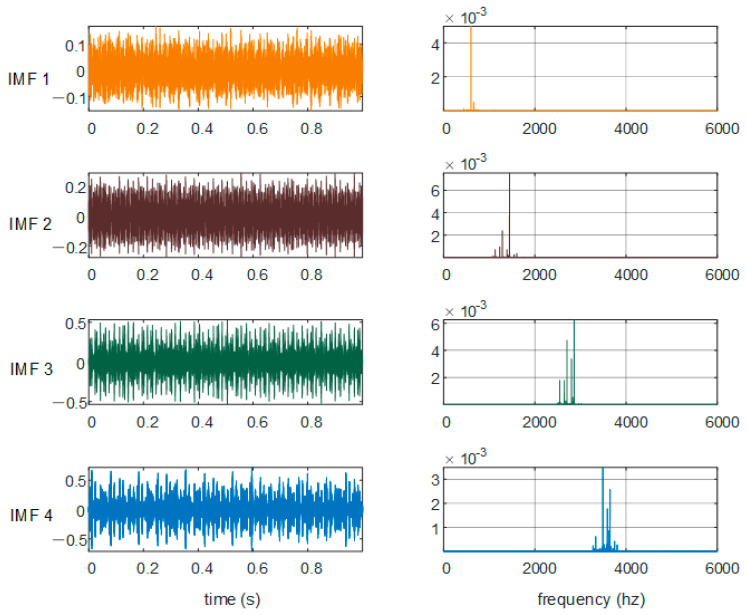
*K* = 4 result of VMD of IR.

The main parameters of VMD are the decomposition number *K* and penalty factor *α*. If the *K* value is too small, there will be mode aliasing or mode loss. If the *K* value is too large, there will be over decomposition. Taking the inner ring fault with a fault size of 0.014 inch as an example, the penalty factor is set to 2000, and the optimal *K* value is obtained by the center frequency observation method [26]. Figure 3 shows the center frequency curves when *K* is equal to 3, 4, 5, and 6, respectively, in the VMD iteration process. It can be seen from Figure 3 that when *K* = 5 or 6, the two curves move closer, which means mode aliasing. When *K* = 4, the center frequency curves of each modal component do not affect each other. Meanwhile, combined with the inner ring fault time domain diagram and frequency domain diagram given in Figure 4, there is no modal aliasing phenomenon, so *K* = 4 was selected in this paper.

Lower entropy values lead to lower dispersion degrees of the data. The modal component with a minimum entropy value in each fault state signal is selected for subsequent research. The entropy values of modal components of each fault type are shown in Table 2. The minimum modal components of each fault type are selected and highlighted in bold. The data of seven fault types are selected respectively: IMF3, IMF4, IMF4, IMF4, IMF4, IMF3, and IMF4.

### 4.3. Feature Extraction by RCMDE

**Figure 5:**
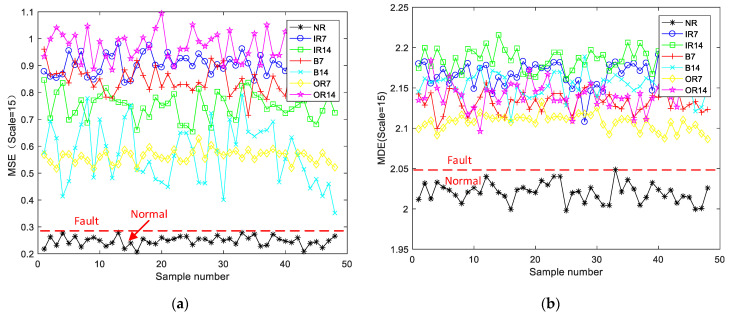

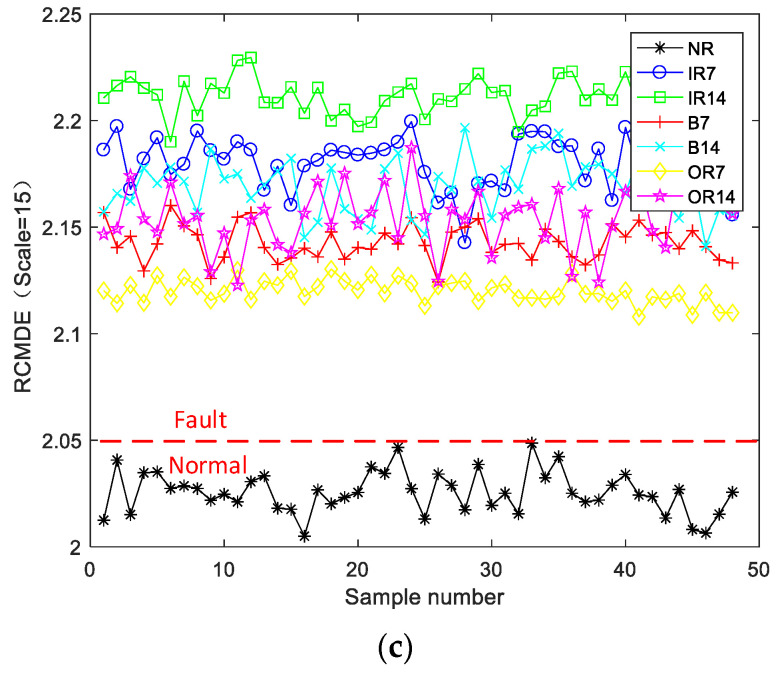
Entropy distribution of all samples (scale = 15): (**a**) MSE; (**b**) MDE; (**c**) RCMDE.

MSE, MDE, and RCMDE were used to extract the features of the best modal components selected in the previous section and then compared, as shown in Figure 5. Compared with Figure 5a,b, the MDE method is more discriminative than the MSE method, so it was chosen for feature extraction. Further comparison shows that the three methods can identify whether the bearing is faulty. However, when MDE and MSE are used for feature extraction, the extracted fault signals overlap and crisscross each other, so it is difficult to distinguish between fault types. When RCMDE is used for feature extraction, the entropy distribution curve has better discrimination, so the extraction effects are enhanced. However, RCMDE can only distinguish two fault types, so it is necessary to use a classifier to classify the data.

**Figure 6:**
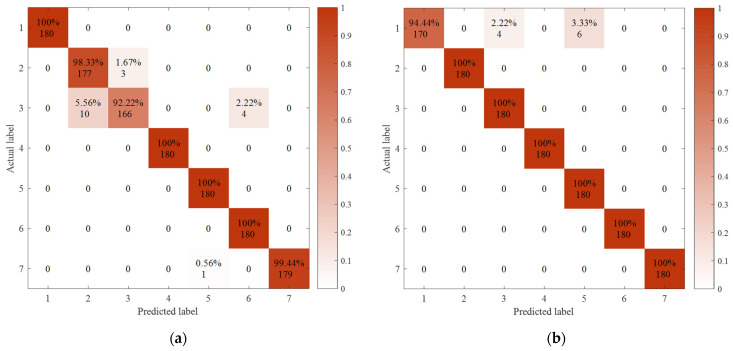

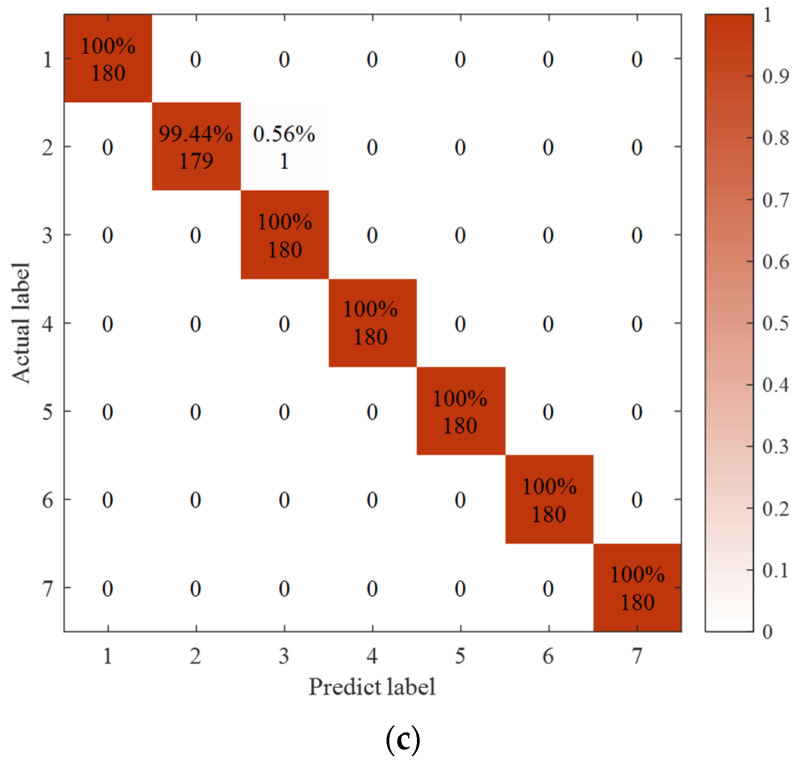
The confusion matrix of different feature-extraction methods: (**a**) MSE; (**b**) MDE; (**c**) RCMDE.

**Figure 7:**
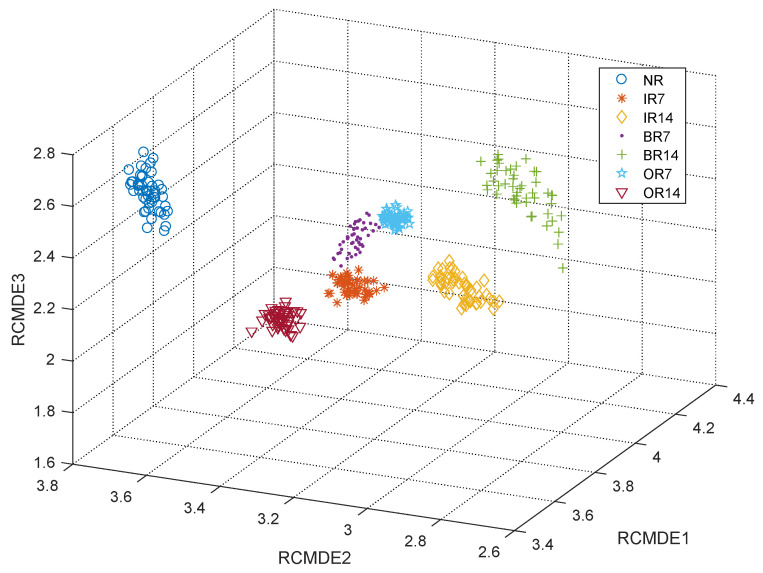
3D projection of feature vectors for different faults.

To further verify the superiority of RCMDE in feature extraction, the first five scale-feature vectors extracted by MSE, MDE, and RCMDE were inputted into the SVM classifier for classification. The classification accuracy and calculation time were recorded. To reduce the experimental error, the average values of 10 experiments were taken, and the results are shown in Table 3. It can be seen from the table that the feature-extraction effect of RCMDE is the best. Its classification accuracy is 99.92%. Combined with the confusion matrix of different feature extraction methods in Figure 6, it further verifies that RCMDE achieves good results. When processing noisy signals, the composite multiscale entropy can improve the stability of entropy results [27]. Under actual working conditions, the bearing is affected by the factors such as fitting accuracy, component damage, and impurity pollution, so noise and vibration often exist at the same time. Therefore, RCMDE is selected to extract fault features. As shown in Figure 7, the feature vectors of the first three scales extracted by RCMDE were taken to generate a 3D projection, and it was found that the fault types were well-differentiated.

### 4.4. Parameter Setting of SSA

The parameters of SSA are set in Table 4. After SSA optimization, the penalty parameter *c* of SVM is 7.3559, and the kernel function parameter *σ* is 1.2238.

### 4.5. Results and Discussion in CWRU Bearing Dataset

To verify the effectiveness of the proposed fault-classification model, the first five scales of RCMDE were selected as feature vectors and inputted into ELM, SVM, and SSA-SVM. The classification accuracy and CPU times were recorded. Among them, there were 48 samples in each state, a total of 336 samples. Each group of samples randomly selected 30 samples as the training set, and the remaining 18 samples were utilized as the test set. The kernel function of SVM is the radial basis function [28].

**Figure 8:**
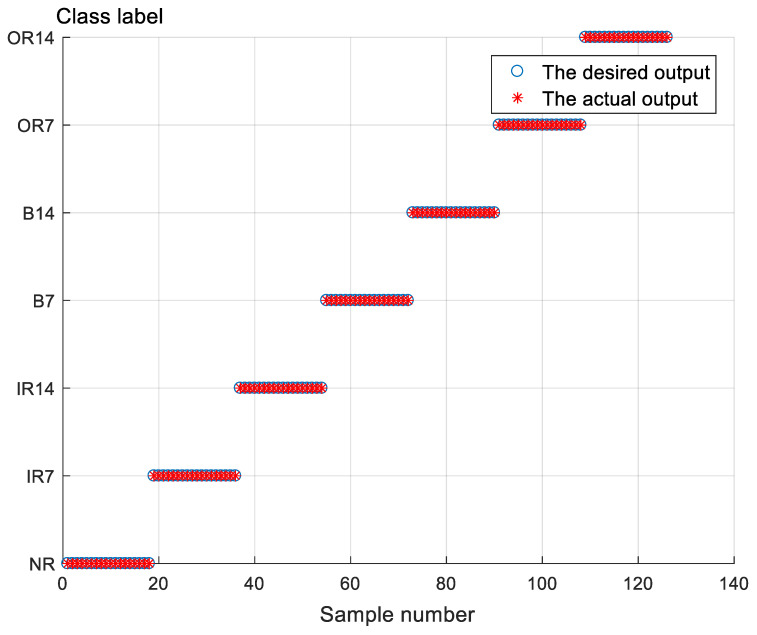
Classification results of the presented method in data sets: CWRU.

The classification results of various models are shown in Table 5. To reduce the influence of randomness, the experiments were repeated 10 times. The results show that the classification accuracy of the proposed method is 100%. Moreover, after SSA optimization, the CPU times of SVM were significantly reduced. As shown in Figure 8, the proposed method can distinguish between seven types of bearing faults.

## 5. Experimental Verification

### 5.1. Bearing Vibration Experiment of WTDS

**Figure 9:**
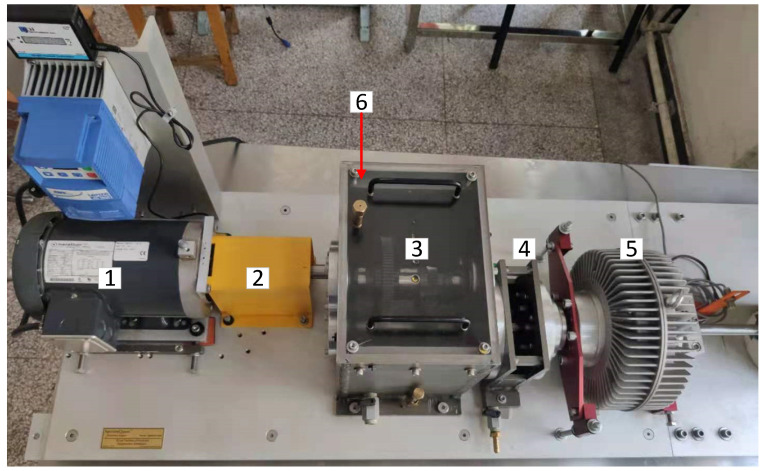
Wind turbine drivetrain diagnostics simulator (WTDS). 1—variable speed drive; 2—torque transducer and encoder; 3—parallel shaft gearbox; 4—planetary gearbox; 5—programmable magnetic brake; 6—radial bearing loader.

To verify the universality of this bearing-fault-classification model, the vibration experiment on the rolling bearings was carried out using the WTDS. The acceleration sensor was installed on the bearing seat to collect the acceleration data in the radial and vertical direction. The structure of the WTDS is shown in Figure 9. The acceleration sensor model is PCB 333b40, and its relevant parameters are shown in Table 6.

**Figure 10:**
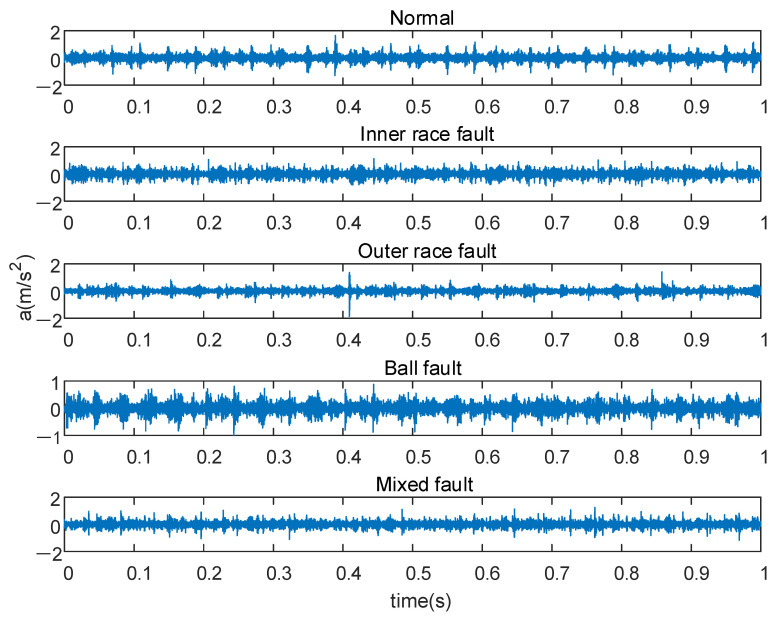
Original vibration signals.

In this experiment, the deep-groove ball bearing of parallel shaft gearbox is selected as the test object. The load current is 0.8 A, the motor speed is 1500 rpm, the sampling frequency is 20,480 Hz, and the sampling time is 5 s. The vibration data of bearings under five working conditions were collected, including one healthy bearing and four faulty bearings. The fault types include inner race fault, outer race fault, ball fault, and mixed fault. Fifty groups of data were collected in each state, and each group contained 2048 sampling points. Then the 250 sets of data are divided into training sets and test sets. Each group of samples randomly selected 30 samples as the training set, and the remaining 20 samples were utilized as the test set. In total, the training set contained 150 groups of data, and the test set contained 100 groups of data. The original vibration signal of the first 1 s is shown in Figure 10. For these five kinds of fault data, we gave them different classification labels according to different properties of bearings: NOR, IF, BF, OF, MF.

### 5.2. Results and Discussion in Bearing Vibration Experiment of WTDS

**Figure 11:**
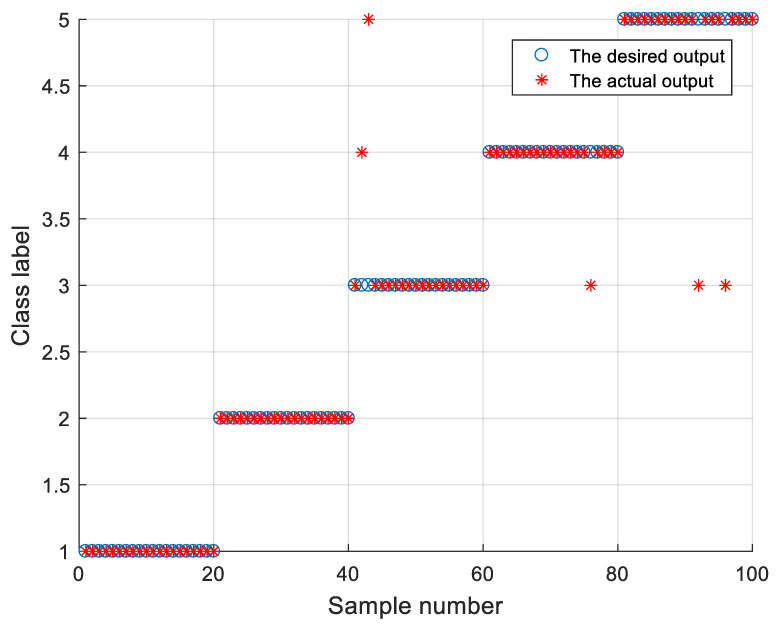
Classification results of the data collected by the WTDS.

The method proposed in this paper was used to classify the data collected by the WTDS. To reduce the influence of randomness, the experiments were repeated 10 times. The results are shown in Table 7 and Figure 11. The effect of fault classification is better than that achieved by other methods, which shows that the proposed method is universal and effective. We noticed that the performance of the classification model will drop with a change in working conditions [29]. The working conditions of the laboratory are different from those of CWRU, and the accuracy of the classification model cannot reach 100%. However, the proposed method still achieves good classification accuracy and is superior to other methods.

## 6. Conclusions

To identify different fault states of rolling bearing accurately and efficiently, a novel fault-classification model for rolling bearing was proposed by integrating VMD, RCMDE, and SSA-optimized SVM. Experimental analysis shows that the method has excellent performance. The main results and innovation of this paper are as follows:

(1) A new method of rolling-bearing-fault diagnosis was proposed. SSA was innovatively applied to optimize the parameters of SVM. Through experimental analysis and comparison, it was proven that this method not only identifies bearing-fault types quickly and effectively, but also has better performance than other similar methods.
(2) Through experimental analysis, it is proved that the fault feature-extraction method based on VMD and RCMDE can fully mine the fault information.
(3) The vibration experiment of rolling bearing is carried out using a WTDS to collect the acceleration signal, which further proves the effectiveness and universal of the proposed method.

In the future, we will further study entropy theory and apply a similar method to the fault diagnosis of planetary gear box and other mechanical equipment. At the same time, we will realize the fault diagnosis method of webpages via programming and combine that information with the former project, and ultimately realize the integration of signal acquisition, signal transmission, and fault diagnosis. The proposed method should be robust when faced with changes of working conditions or unseen conditions during the training of the model. In the future research, we will explore a reliable method to improve the generalization and robustness of the classification model.

## Figures and Tables

**Table 1:** Details of the experimental data.

Fault Type	Image	Classification Label	Fault Size (Inches)	Training Sample Number	Testing Sample Number
Normal	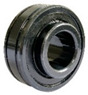	NR	0	30	18
Inner race fault	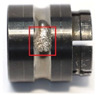	IR7	0.007	30	18
		IR14	0.014	30	18
Ball fault	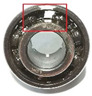	B7	0.007	30	18
		B14	0.014	30	18
Outer race fault	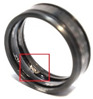	OR7	0.007	30	18
		OR14	0.014	30	18

**Table 2:** The entropy of IMF_1_–IMF_4_.

Classification Label	IMF_1_	IMF_2_	IMF_3_	IMF_4_
NR	3.9651	3.1130	**2.0256**	2.1682
IR7	3.8240	2.7877	2.2784	**2.1812**
IR14	4.3341	2.7363	2.2760	**2.2113**
B7	4.2778	3.1388	2.2618	**2.1427**
B14	4.2357	2.7653	2.2930	**2.1689**
OR7	3.9152	2.1466	**2.1199**	2.1342
OR14	4.1615	3.2886	2.1772	**2.1546**

**Table 3:** Classification results of various entropies.

Entropy Method	Classification Algorithm	Accuracy (%)	CPU Time of Classification (s)
MSE	SVM	98.57	74.51
MDE	SVM	99.05	74.11
RCMDE	SVM	99.92	75.27

**Table 4:** Parameters of SSA.

Population Size	Maximum Number of Iterations	Proportion of Discoverers	Proportion of Watchmen	Security Threshold	Search Range of Parameter *c*	Search Range of Parameter *σ*
10	20	70%	20%	0.6	[1100]	[1100]

**Table 5:** Classification results of different methods.

Different Methods		Accuracy (%)		Standard Deviation	CPU Time of Classification (s)
	Max	Min	Mean		
VMD+MDE+SVM	99.21	98.41	99.05	0.25	74.11
VMD+RCMDE+SVM	100	99.76	99.92	0.09	75.27
VMD+RCMDE+ELM	99.21	96.83	98.41	0.87	1.11
The proposed method	100	100	100	0	3.78

**Table 6:** Sensor parameters.

Sensor Model	Sensitivity	Frequency Range	Temperature Range	Weight
PCB 333B40	500 mV/g	0.5 Hz~3 kHz	−18~+66 °C	7.5 g

**Table 7:** Classification results of the data collected by the WTDS.

Different Methods		Accuracy (%)		Standard Deviation	CPU Time (s)
	Max	Min	Mean		
VMD+MDE+SVM	83.0	74.0	78.8	3.75	53.5
VMD+RCMDE+SVM	92.0	88.0	89.9	1.40	54.8
VMD+RCMDE+ELM	93.0	88.0	89.7	1.42	2.2
The proposed method	96.0	94.0	95.0	0.77	3.7

## Data Availability

https://csegroups.case.edu/bearingdatacenter/home (accessed on 1 March 2021).
